# Advances in graft-versus-host disease: emerging therapeutic strategies, biomarker discoveries, and innovative treatment approaches

**DOI:** 10.3389/fimmu.2026.1816067

**Published:** 2026-06-09

**Authors:** Yilei Cui, Chun-Chun Gau, Chuang-Wei Wang, Chun-Bing Chen, Wen-Hung Chung, Gary J. Fisher, Sung Won Choi

**Affiliations:** 1 Department of Dermatology, University of Michigan Medical School, Ann Arbor, MI, United States; 2 Division of Pediatric General Medicine, Department of Pediatrics, Chang Gung Memorial Hospital, Taoyuan, Taiwan; 3 Department of Dermatology, Drug Hypersensitivity Clinical and Research Center, Chang Gung Memorial Hospital, Linkou, Taipei and Keelung, Taiwan; 4 College of Medicine, Chang Gung University, Taoyuan, Taiwan; 5 Michigan Medicine, Blood and Marrow Transplantation Program and Rogel Cancer Center, Ann Arbor, MI, United States

**Keywords:** acute GVHD, biomarkers, chronic GVHD, cytokines, graft-versus-host disease (GVHD), hematopoietic stem cell transplantation (HSCT), mesenchymal stromal cells (MSCs), pathogenisis

## Abstract

Graft-versus-host disease (GVHD) remains a leading cause of non-relapse mortality after allogeneic hematopoietic stem cell transplantation (HSCT). This review provides a concise overview of the clinical spectrum of GVHD, including classical acute GVHD (aGVHD), chronic GVHD (cGVHD), and overlap syndromes as defined by the NIH Consensus Criteria. We summarize key advances in precision diagnostics, including the ST2/REG3α MAGIC biomarker panel, novel B-cell subsets (cGPS), which derive from a CD27+CD86+CD20- B-cell subpopulation, and transcriptomic profiling using single-cell RNA sequencing (scRNA-seq) and plasma cell-free RNA (cfRNA). We also examine extracellular vesicles and loss of gut microbiota diversity as critical drivers of pathogenesis. Finally, we analyze the current therapeutic landscape, distinguishing between 2024 prophylaxis recommendations and newly approved treatments for steroid-refractory disease.

## 1 Introduction

Allogeneic hematopoietic stem cell transplantation (HSCT) is a potentially curative therapy for hematologic malignancies and selected non-malignant disorders, offering the possibility of durable remission or cure (1, 2). The procedure combines a preparative conditioning regimen to eradicate diseased or dysfunctional hematopoiesis with the infusion of donor-derived stem cells that engraft and reconstitute the recipient’s hematopoietic and immune systems. In the United States, an estimated 7, 024 allogeneic HSCTs were performed in adult patients in 2015, with 1, 304 procedures in pediatric patients, highlighting its broad clinical utilization (3). More recent data from the Center for International Blood and Marrow Transplant Research (CIBMTR) indicate that transplant volumes have continued to rise, reflecting the expanding use of this potentially curative modality.

A major limitation to allogeneic HSCT is graft-versus-host disease (GVHD), an immune-mediated complication in which donor-derived immune cells recognize recipient antigens and damage host tissues (4). Acute GVHD classically involves the skin, gastrointestinal tract, and liver, but can evolve into a chronic, multisystem inflammatory and fibrosing syndrome with substantial long-term morbidity (5). Contemporary clinical practice distinguishes acute and chronic GVHD using NIH Consensus Criteria rather than an arbitrary day-100 cutoff, yet diagnostic and risk-stratification challenges persist due to clinical overlap with infection, drug toxicity, and other post-transplant inflammatory syndromes (6).

### 1.1 Clinical burden and unmet need

Beyond direct tissue injury, GVHD imposes a substantial clinical burden after allogeneic HSCT by driving severe organ dysfunction, prolonged immunosuppression, infectious complications, impaired quality of life, and non-relapse mortality (7). Acute GVHD and its treatment contribute to infectious complications and early mortality after HSCT (8). Even when not immediately fatal, GVHD frequently necessitates prolonged systemic immunosuppression, which increases susceptibility to bacterial, viral, and fungal infections and contributes to recurrent hospitalizations and long-term functional impairment, particularly in chronic GVHD (9). While PTCy-based prophylaxis has reduced GVHD rates in contemporary transplant settings, GVHD remains a clinically significant complication and a key upstream driver of infectious morbidity and non-relapse mortality (10).

Epidemiologically, GVHD affects approximately 40%–60% of HSCT recipients, with roughly one-third developing higher-grade acute GVHD (grade III–IV), which is consistently associated with worse outcomes (11). Severe cutaneous presentations, including SJS/TEN-like acute GVHD, are associated with particularly poor short-term survival and may reach very high mortality during follow-up (12). Reflecting its downstream impact on post-transplant outcomes, 2025 CIBMTR data of U.S. allogeneic HSCT recipients (2019–2023) attribute a measurable proportion of deaths to GVHD, with differences by age and timing: in pediatric patients, GVHD accounts for 4% of deaths within the first 100 days and 6% at or beyond day 100, while in adults GVHD accounts for 9% of deaths both within and beyond the first 100 days post-transplant (13).

### 1.2 Scope of this review

This review provides a focused, clinically oriented synthesis of: (i) GVHD immunopathogenesis across acute and chronic phenotypes; (ii) advances in prognostic and diagnostic biomarkers spanning protein panels (e.g., ST2/REG3α), cellular and B-cell subset profiling, transcriptomics (including single-cell RNA sequencing), and plasma cell-free RNA; (iii) emerging contributors to GVHD biology and risk, including extracellular vesicles and intestinal microbiota dynamics; and (iv) the evolving therapeutic landscape, distinguishing contemporary prophylactic strategies and treatments for steroid-refractory disease.

### 1.3 Gaps in existing literature

Despite progress, several gaps limit the translation of mechanistic and biomarker discoveries into routine care. Many proposed biomarkers and multi-omic signatures lack harmonized pre-analytic workflows, cross-platform reproducibility, and prospective validation across diverse donor sources, conditioning intensities, and prophylaxis platforms, complicating generalizability and clinical implementation (14). Mechanistic frameworks increasingly recognize contributions from endothelial injury, tissue-resident immunity, extracellular vesicle signaling, and microbiota-derived metabolites, but causal pathways, temporal ordering, and actionable therapeutic nodes remain incompletely defined in humans (15). Although newer prophylaxis regimens (including PTCy-based approaches) and targeted agents for treatment have improved outcomes for subsets of patients, steroid-refractory disease and chronic GVHD continue to drive morbidity, infectious risk, and non-relapse mortality, highlighting the need for earlier risk identification, treatment personalization, and steroid-sparing strategies (6).

### 1.4 Organization of the review

We first summarize the current understanding of GVHD pathophysiology and the immune cascades that underline tissue injury. We then review established and emerging biomarkers, followed by genetic/epigenetic determinants and transcriptomic and miRNA-based signatures. Next, we discuss extracellular vesicles and the intestinal microbiota as modulators of GVHD risk and severity. Finally, we outline contemporary approaches to GVHD prevention and treatment, emphasizing recent advances and remaining therapeutic challenges.

## 2 Pathophysiology of GVHD

The immunopathogenesis of GVHD is orchestrated through a multifaceted cascade of alloimmune activation, in which donor T cells initiate a cytotoxic response against host antigen-presenting cells (16). Regulatory T cells (Treg), specifically CD4+CD25 high Treg, express the master regulatory transcription factor FOXP3, which is being studied for GVHD prevention and therapy (16). The immunopathogenesis of GVHD is best understood through an established three-phase cascade that meets Billingham’s seminal criteria for disease (17). This cascade is modulated by counter-regulatory mechanisms, including Tregs and myeloid-derived suppressor cells (MDSCs), which attempt to restore the balance between immune activation and tolerance.

The classical foundational three-phase model of aGVHD, while integrating recent mechanistic advances (16).

- Phase I (Afferent phase): Conditioning-induced tissue injury, particularly in the gastrointestinal tract, releases damage-associated molecular patterns (DAMPs; e.g., ATP) and promotes translocation of pathogen-associated molecular patterns (PAMPs; e.g., lipopolysaccharide [LPS]) (16, 18). These signals activate host antigen-presenting cells (APCs) and trigger production of pro-inflammatory cytokines, including tumor necrosis factor-α (TNF-α), interleukin (IL)-1, and IL-6 (18).
- Phase II (Central phase): Host APCs present alloantigens to donor T cells, inducing their activation and expansion (16). Tissue-specific homing molecules, particularly integrin α4β7, direct alloreactive T cells to the intestinal mucosa and contribute to organ-specific GVHD (19).
- Phase III (Efferent phase): Effector T cells, natural killer (NK) cells, and activated myeloid cells mediate tissue injury through Fas/FasL ligand and perforin/granzyme pathways (16). Macrophage-derived inflammatory mediators, including nitric oxide (NO), further impair epithelial stem cell proliferation and delay tissue repair, especially in the gastrointestinal tract (20, 21).

Pathophysiology: new findings and emerging concepts.

Recent work has refined the canonical three-phase model by identifying tissue-resident and non–T-cell pathways that can independently amplify or sustain acute GVHD, while also clarifying why gastrointestinal involvement is both common and prognostically dominant. Conditioning injury and early inflammatory cues disrupt intestinal stem cell programs and Paneth cell function, reducing antimicrobial peptide production and compromising barrier integrity (22). Barrier disruption promotes microbial translocation and fuels a self-reinforcing loop of epithelial injury, innate immune activation, and impaired regeneration (23).

The microbiome has emerged as an important determinant of acute GVHD risk, severity, and treatment response. Loss of intestinal microbial diversity after HSCT, antibiotic-driven dysbiosis, and depletion of commensals that generate short-chain fatty acids have been linked to impaired epithelial repair and heightened inflammatory signaling (24). These findings have shifted attention toward microbiome-sparing antimicrobial strategies and microbiota-restoring interventions (for example, rationally designed consortia in selected settings) as potential adjuncts to standard immunosuppression (25). Furthermore, bile acid metabolism and microbial metabolites are increasingly recognized as immune-modulating signals that influence epithelial integrity and myeloid activation, providing plausible mechanistic links between supportive care practices, gut ecology, and acute GVHD outcomes (26).

The field has expanded beyond a strictly T cell-centric view, implicating donor-derived macrophage-like cells as key mediators of tissue injury. These cells have been shown to predominate in GVHD tissue, to express genes associated with antigen presentation and costimulation pathways, and to promote allogeneic T cell activation and inflammatory cytokine production (27). Neutrophil extracellular traps and complement activation have also been implicated as amplifiers of tissue injury and vascular inflammation in experimental and translational studies (28). Moreover, a recent review on GVHD T-cell metabolism suggests that alloreactive T cells may undergo distinct metabolic reprogramming in target organs, and that mTOR, glycolysis, and related pathways shape effector behavior (29). Thus, strategies that promote tolerance, for example, T-reg enhancing approaches, are being developed in current clinical trials (30).

Finally, major advances in translational profiling and biomarker science are changing how acute GVHD pathophysiology is studied and, increasingly, how risk is conceptualized clinically (31, 32). Large multicenter efforts have validated plasma biomarker panels that reflect epithelial injury and GI tract involvement and can identify high-risk disease early, often before irreversible tissue damage is clinically apparent (33). Single-cell and spatially resolved technologies applied to patient tissues are now mapping the cellular neighborhoods of aGVHD lesions, revealing coordinated interactions among epithelial cells, endothelium, myeloid cells, and distinct T-cell states. Together, these emerging concepts support a more integrated model of acute GVHD in which epithelial and endothelial injury, innate immune amplification, and microbiome-derived signals shape the magnitude and tissue distribution of alloimmune damage (22), providing a rationale for biomarker-guided therapy and for interventions that combine targeted immunomodulation with strategies that preserve barrier function and reduce infectious complications.

## 3 Biomarkers associated with GVHD prognosis

Biomarkers that play an increasingly critical role in early diagnosis and in predicting the prognosis of GVHD are listed in **Table 1**, which includes specific proteins, cytokines, and metabolites.

**Table 1:** Potential prognostic and diagnostic biomarkers in GVHD.

Biomarker	Source(s)	Potential clinical value	Acute/ Chronic	Clinical associations
CXCL-9 (45, 46, 122, 123)	Plasma	Prognosis	aGVHD cGVHD	Elevated in newly diagnosed
Dickkopf-3(DKK3) (46)	Plasma	Prognosis	cGVHD	Elevated predicts future disease occurrence
Elafin (36, 38, 39)	Plasma/Skin	Diagnosis/Prognosis	aGVHD	Elevated correlations with skin involvement and severity
Galectin-3 (Gal3) (48)	Plasma	Diagnosis	aGVHD	Elevated in severe grading (grades 2-4)
Glycochenodeoxycholate, Pyridoxate (47)	Blood metabolites	Protective	aGVHD	Elevated in severe grading (grades 3-4)
Interleukin-2, -4 (IL-2, IL-4) (44)	Serum	Prognosis	aGVHD	Elevated rapidly reflects early, strong pro-inflammatory immune activation and donor T cell responses
IL-6 (42)	Plasma	Prognosis	aGVHD	Elevated in severe grading (grades 3-4)
MMP3 (46)	Plasma	Prognosis	cGVHD	Elevated predicts future disease occurrence
Osteopontin (OPN) (43)	Plasma	Diagnosis/Prognosis	aGVHD	Increased secretion specifically from CD4+ T cells has a protective effect by preventing intestinal epithelial cell death
Regenerating Islet-derived Protein 3-α (REG3α) (35)	Plasma	Diagnosis/Prognosis	aGVHD	Elevated predicted gastrointestinal tract damage
Suppression of Tumorigenicity 2 (sST2) (35)	Plasma	Diagnosis/Prognosis	aGVHD	Elevated predicted gastrointestinal tract damage and mortality
Soluble Vascular Cell Adhesion Moleculer-1(sVCAM-1) (40)	Plasma	Prognosis	aGVHD	Elevated in severe grading (grades 2-4)
TGF*-β* (44)	Serum	Prognosis	aGVHD	Decrease in severe grading
T cell Ig and Mucin Domain 3 (TIM3) (42)	Plasma	Prognosis	aGVHD	Elevated in severe grading
Toll-like Receptor 4(TLR4) (44)	Serum	Prognosis	aGVHD	Elevated in severe grading (grades 3-4)
Soluble Tumor Necrosis Factor Receptor 1(TNFR1) (42)	Serum	Prognosis	aGVHD	Elevated in severe grading (grades 3-4)
von Willebrand Factor (VWF)and factor VIII (FVIII) (41)	Plasma	Diagnosis	aGVHD	Elevated reflects endothelial dysfunction

### 3.1 Tissue damage biomarkers

Some tissue-damage biomarkers, including suppression of tumorigenicity 2 (ST2), regenerating islet-derived protein 3-α (REG3α), and Elafin, have been shown to predict GVHD (34–38). The combination of ST2 and REG3α, known as the MAGIC (Mount Sinai Acute GVHD International Consortium) biomarker panel, is the most widely validated tool for predicting aGVHD severity and mortality in adults. While the MAGIC Algorithm Probability (MAP) score has shown promise in pediatric cohorts, its widespread routine clinical implementation remains an area of ongoing investigation (35). Elafin is recognized as a specific biomarker of skin aGVHD, as it is overexpressed in inflamed epidermal tissues (36, 38, 39). Furthermore, markers of endothelial damage, reflecting systemic vascular injury, include soluble vascular cell adhesion molecule-1 (sVCAM-1), von Willebrand factor (vWF), and factor VIII (FVIII) (40, 41). Other key markers reflecting severity and mortality risk include T cell Ig and mucin domain 3 (TIM3) and soluble tumor necrosis factor receptor 1 (sTNFR1) (42). In contrast to most inflammatory markers, osteopontin, specifically the secreted isoform, has been found to be protective in aGVHD models. It has been shown to act on the CD44 receptor expressed on intestinal epithelial cells to abate cell death (43).

### 3.2 Endothelial and inflammatory markers

Several inflammatory cytokines and receptors also reflect the systemic immune responses characteristic of GVHD. One study reported elevated concentrations of Interleukin-2 (IL-2), Interleukin-4 (IL-4), and Interleukin-6 (IL-6) in aGVHD patients (44). Notably, Toll-like receptor 4 (TLR4) was found to be significantly increased in patients with severe (Grade III/IV) aGVHD and in steroid-refractory patients (44). Chemokine (C-X-C motif) ligand 9 (CXCL9) is a potent chemokine that is consistently elevated at the onset of *de novo* childhood GVHD diagnosis (45). Dickkopf-3 and matrix metalloproteinase-3 (MMP3) are critical markers associated with the fibrotic components of cGVHD (46).

### 3.3 Metabolic and cell-specific markers and others

In addition, recent studies have established causal links between certain metabolites and GVHD risk. Pyridoxate, a derivative of Vitamin B6, was identified as a protective factor against Grade 3–4 aGVHD by increasing the level or function of CD39+ Treg (47). Conversely, Glycochenodeoxycholate, a bile acid derivative, was identified as a causal risk factor for Grade 3–4 aGVHD (47). Elevated levels of Galectin-3, a lectin involved in T cell regulation and inflammation, are associated with increased severity of GVHD (48). It is essential to distinguish between aGVHD and cGVHD biomarkers (49). For instance, Galectin-3 predicts aGVHD outcomes, whereas CXCL9 is a specific biomarker for cGVHD. Recent research has further identified the cGVHD Progress Score (cGPS), derived from a CD27+CD86+CD20− B-cell subpopulation (50), as a novel tool for monitoring disease progression.

## 4 Genetic and epigenetic factors associated with GVHD prognosis

The HLA genes, located on chromosome 6, encode highly polymorphic proteins that are integral to immune surveillance and antigen presentation (51, 52). In the context of allogeneic HSCT, HLA compatibility between the donor and recipient is paramount for reducing the incidence and severity of GVHD (53). The HLA system is broadly classified into class I (HLA-A, -B, -C) and class II (HLA-DR, -DQ, -DP) molecules, both of which contribute to immune recognition (52, 54). Disparities in HLA alleles can provoke alloreactivity, a phenomenon in which donor T cells recognize recipient HLA molecules as non-self, thereby initiating a cascade of immune responses that culminate in GVHD (52, 55). The severity of GVHD is primarily determined by the number and nature of these HLA mismatches. For instance, patients with HLA-B62 positivity have shown a notably reduced risk of transplant-related mortality compared to HLA-B62-negative patients (56). Conversely, HLA-B60-positive patients face a substantially elevated risk of mortality compared with their negative counterparts (56). These findings highlight the prognostic significance of specific HLA alleles in HSCT outcomes.

Despite optimal HLA matching, GVHD can still occur due to minor histocompatibility antigens (53, 55). Genetic variations in minor histocompatibility antigens (mHAs) between the donor and recipient can result in T-cell recognition and subsequent GVHD (53). Importantly, minor histocompatibility antigens can also contribute to the graft-versus-leukemia (GVL) effect, whereby donor-derived T cells recognize and eradicate leukemic cells in the recipient. Thus, balancing GVHD and GVL represents a critical challenge in HSCT. However, these same HLA and mHA disparities form the immunological basis for the graft-versus-leukemia (GvL) effect. The fundamental tension between maximizing GvL to prevent relapses while minimizing GVHD is a central challenge in HSCT (53). Genetic variations in cytokine genes have also been implicated in modulating GVHD susceptibility and severity. Polymorphisms in cytokine-related genes, such as IL1RL1, have been identified as pre-transplant biomarkers for GVHD risk and infection-related mortality (57). Additionally, interactions between killer-cell immunoglobulin-like receptors (KIRs) on natural killer (NK) cells and HLA molecules further influence post-transplant immune responses. KIR-HLA interactions are known to regulate NK cell activity, thereby modulating the risks of both GVHD and GVL (58). Moreover, soluble MHC class I-related chain concentrations have been identified as key determinants in GVHD pathogenesis following allo-HSCT (59).

Recent advances in molecular diagnostics have expanded the potential for using cell-free DNA (cfDNA) as a biomarker for GVHD and other post-transplant complications. Studies utilizing low-coverage bisulfite sequencing have demonstrated that cfDNA profiling can provide valuable insights into transplant-related complications, including GVHD, infection, relapse, and graft failure (60). By analyzing methylation markers, researchers have found that patients with active cGVHD exhibit elevated cfDNA levels and tissue-specific methylation signatures that correlate with clinical disease severity (61). Furthermore, increased mitochondrial cfDNA levels have been observed in patients undergoing HSCT with a significant correlation to the presence of cGVHD (62). These findings collectively support the emerging role of cfDNA as a biomarker with the potential to improve GVHD diagnosis, disease monitoring, and therapeutic stratification in post-transplant care.

## 5 MicroRNA and gene expression profiles associated with GVHD prognosis

### 5.1 MicroRNAs (miRNAs) have emerged as potential biomarkers for aGVHD, offering potential advantages in prediction, diagnosis, and disease monitoring

One study that evaluated circulating miRNAs in lymphoma patients following allogeneic HSCT from matched unrelated donors identified significant upregulation of miR-194 and miR-518f in aGVHD samples compared to non-aGVHD controls (63). This finding suggests that specific miRNA signatures could serve as early indicators of disease onset. Furthermore, research on cutaneous aGVHD has identified miRNAs involved in inflammation, tissue damage, cellular proliferation, and tissue repair (64). A specific miRNA panel, including miR-30a, miR-93*, miR-142, miR-155, miR-199a-3p, miR-377, and miR-423, has been associated with these pathological processes, highlighting the potential of miRNA profiling as a diagnostic tool (65–67). Stickel et al. demonstrated that host miR-146a deficiency exacerbates GVHD by modulating dendritic cell function through the JAK-STAT signaling pathway, further supporting the regulatory role of miRNAs in GVHD pathogenesis (68). Collectively, these findings support the utility of miRNAs as biomarkers for GVHD; however, further research is needed to clarify the molecular mechanisms and functional roles in disease progression (69).

### 5.2 Single-cell RNA sequencing (scRNA-seq) has emerged as a powerful tool for dissecting cellular heterogeneity and dynamic changes in immune cell populations during GVHD

This approach has been instrumental in characterizing mononuclear phagocytes in cutaneous GVHD, revealing an expansion of CD163+ tissue-resident macrophages with anti-inflammatory and tissue-remodeling properties (70). scRNA-seq of CD4+ T cells at Day 100 post-transplant has identified an interferon-driven inflammatory CD4 naïve T-cell subpopulation that predicts moderate-to-severe cGVHD (71), enabling pre-symptomatic risk stratification. Additionally, liquid biopsy via plasma cell-free RNA (cfRNA), specifically the CCL21/RHD ratio, captures post-transplant immune dynamics and predicts GVHD development (72). Complementary methodologies, such as immunofluorescence, have been employed to validate scRNA-seq findings, reinforcing the observed expansion of CD163+ macrophages in aGVHD skin lesions (70) (73). These studies highlight the important contributions of scRNA-seq in advancing our understanding of GVHD pathogenesis and in identifying potential therapeutic targets for disease modulation. Spatiotemporal analysis of donor CD8+ T cells has further delineated the mechanisms of tissue infiltration and destruction during gastrointestinal aGVHD, highlighting the critical role of T cell subsets in disease development and progression. Furthermore, spatial transcriptomics has revealed distinct tissue niches in the GI tract linked to steroid responsiveness (22, 31).

## 6 Extracellular vesicles associated with GVHD prognosis

Extracellular vesicles (EVs) have gained considerable attention for their roles in intercellular communication and their implications in both the pathogenesis and treatment of GVHD. EVs including exosomes, microvesicles, and apoptotic bodies, are membrane-bound particles that facilitate intercellular communication. In GVHD, T-cell-specific EVs and those carrying sVCAM-1 serve as potential monitoring tools. EVs are a heterogeneous group of lipid bilayer-enclosed membranous vesicles, ranging from the nano- to micrometer scale, that are actively secreted or shed by cells into the extracellular environment (74). Based on their biogenesis and release mechanisms, EVs are primarily classified into three main categories: exosomes (formed by the fusion of multivesicular bodies with the plasma membrane), microvesicles (formed by direct budding from the plasma membrane), and apoptotic bodies (released during programmed cell death) (75). Acting as critical mediators of intercellular communication, EVs transfer various bioactive molecules, such as proteins, nucleotides, lipids, and metabolites, from donor to recipient cells, thereby regulating vital biological functions including immune modulation, antigen presentation, and inflammatory responses (76, 77). In the context of allogeneic hematopoietic stem cell transplantation (allo-HSCT), circulating EVs undergo distinctive quantitative and qualitative changes, such as a sudden increase following T cell activation (78). Consequently, these vesicles play a crucial role in the immunological dysregulation underlying GVHD pathogenesis and are also emerging as highly promising diagnostic biomarkers and potential cell-free therapeutics.

Increasing evidence suggests that EVs, particularly those derived from mesenchymal stromal stem cells (MSCs), function as both biomarkers and therapeutic agents in GVHD. The potential of EVs as biomarkers is supported by studies showing their association with GVHD severity and clinical outcome. Clinical observations further support these findings, as exemplified by a case in which MSCs-EVs were used to successfully treat a patient with steroid-refractory aGVHD, leading to symptomatic improvement and a reduction in steroid dependence (79–81). Despite the promising potential of EV-based therapies, the lack of standardized protocols for EV production, dosing, and administration remains a significant barrier (82). The scalability of EV isolation, the reproducibility of therapeutic effects, and the establishment of stringent quality control measures need to be addressed (83). Moreover, standardized reporting of outcomes and meta-analyses is necessary to consolidate findings across studies and facilitate the translation of EV-based therapies into clinical practice. Despite these hurdles, preclinical and clinical studies highlight the potential of EVs, particularly MSCs-EVs, to transform the diagnosis and treatment of GVHD.

## 7 Microbiota

Technological advancements in microbiome research have significantly enhanced understanding of the intestinal microbial flora and their interactions with the host immune system in HSCT patients, as well as of post-transplant complications (24, 25). The commensal microbiome is frequently dysregulated in HSCT patients. HSCT patients frequently experience dysregulation of the commensal microbiome, often because of conditioning regimens, which typically involve chemotherapy, with or without total body irradiation. These regimens can compromise the intestinal barrier, leading to bacterial translocation into host tissues, neutrophil infiltration, and subsequent damage to the barrier. Bacterial translocation can further induce monocyte activation, which promotes T helper 17 (Th17) cell differentiation, resulting in macrophage and neutrophil accumulation at inflammatory sites, as well as dendritic cell activation, ultimately directing alloreactive cytotoxic T cells to target host tissues (23). Additionally, microbiota-derived short-chain fatty acids serve as an energy source for intestinal epithelial cells and play a protective role in GVHD by inducing Treg, which secrete anti-inflammatory cytokines (23).

While the conditioning regimen initiates intestinal mucosal injury, it is also critical to recognize that post-transplant gut microbiome dysbiosis is a multifactorial process profoundly exacerbated by antimicrobial exposure and dietary modifications (84, 85). The administration of broad-spectrum antibiotics, whether used prophylactically or therapeutically, drives severe perturbations of the gut microbiome (84, 85). Concurrently, enforced dietary modifications and the route of nutritional support during the transplant period critically disrupt microbiota homeostasis (84). Transplant-related complications, such as severe mucositis and nausea, often enforce prolonged fasting and necessitate a shift to parenteral nutrition (PN) (84). Exclusive reliance on PN deprives the luminal environment of essential substrates such as dietary fiber, leading to mucosal atrophy, diminished microbial richness, and a marked reduction in short-chain fatty acid (SCFA)-producing bacteria, such as *Blautia* (85, 86). Furthermore, specific dietary components can act as catalysts for dysbiosis; for instance, conditioning-induced intestinal damage often causes transient lactase deficiency, allowing undigested luminal lactose to act as a primary substrate that specifically fuels the expansion and domination of *Enterococcus* species (84, 86). Together, these antimicrobial and dietary interventions, acting independently and cumulatively, compound the initial conditioning injury, leading to a profound and sustained disruption of microbiota homeostasis.

A hallmark feature of post-HSCT dysbiosis is not merely the depletion of specific individual bacterial species, but rather a profound loss of overall intestinal microbial diversity (alpha diversity). This extensive reduction in commensal flora is frequently accompanied by a phenomenon known as intestinal domination, in which a single taxonomic group expands to comprise the vast majority (often ≥30%) of the microbiome (87). Large multicenter analyses and meta-analyses have consistently demonstrated that lower intestinal diversity, particularly during the peri-engraftment period, is a strong, independent predictor of elevated transplant-related mortality (TRM) and inferior overall survival (OS) (84, 88, 89). Furthermore, diminished microbial diversity significantly increases the risk of developing moderate-to-severe (grade II-IV) aGVHD (89).

Conditioning regimens often lead to a profound loss of microbial diversity. A key feature of post-HSCT dysbiosis is the expansion of multi-drug resistant (MDR) bacteria (90), such as Enterococcus spp., which are frequently detected in patients. This loss of overall diversity, rather than the depletion of a single species, serves as a significant predictor of severe GVHD and increased transplant-related mortality.

Longitudinal studies of the microbiome profile may enable early identification of patients at high risk for GVHD, potentially paving the way for interventions such as fecal microbiota transplantation to restore microbiota integrity. Studies have identified distinct patterns of microbial disruption and recovery that may enable preemptive prediction of acute GVHD based on pre-transplant microbiota composition (91). Compared to healthy controls, HSCT patients exhibit distinct microbiome alterations, suggesting that targeted preventive strategies could be developed. Notably, depletion of *Blautia* spp. and expansion of *Enterococcus* spp. in the gut after HSCT have been confirmed, with the precise definition of phylogenetically closely related sequence variants of these genera characteristic of these patterns and of when they return to pre-HSCT levels (92). High abundances of intestinal *P. distasonis* (Tannerellaceae), oral Actinomyces sp. (Actinomycetaceae), and other taxa from different body sites pre-HSCT predict the development of moderate-to-severe acute GVHD post-transplant. Additionally, rapid B and NK cell reconstitution is associated with high abundances of Lachnospiraceae and Ruminococcaceae, which also depend on antibiotic treatment (93). Distinct sequence variants across multiple body sites have been associated with Th17 cell counts, highlighting the need for further research into the immunomodulatory role of the microbiome in inflammation regulation and the development of aGVHD (93). Moreover, shared host–microbial associations across body sites present opportunities to integrate oral and nasal swab sampling into both research and clinical diagnostic workflows (94). These approaches may contribute to the development of precision-based therapeutic strategies to minimize severe post-transplant complications, enhance immune recovery, and restore microbiota homeostasis.

## 8 Proteomics biomarkers of GVHD prognosis

Proteomic approaches have become integral in identifying diagnostic, predictive, and prognostic biomarkers for GVHD. These studies use biofluids, including peripheral blood, urine, and saliva, to identify biomarkers that reflect systemic or localized disease activity. High-throughput proteomic platforms, such as liquid chromatography-tandem mass spectrometry, capillary electrophoresis coupled with mass spectrometry, and enzyme-linked immunosorbent assays, have been instrumental in identifying potential candidate biomarkers. A primary objective of these investigations is to identify biomarkers early, before the onset of clinical symptoms, enabling stratification of patients by risk of severe disease and post-transplant complications (95).

A longitudinal proteomics study has identified potential metabolic markers linked to aGVHD, with retinol-binding protein 4 emerging as a possible indicator of insulin resistance and its association with aGVHD and/or skin GVHD. These findings suggest that insulin resistance and metabolic disturbances may be immediate post-transplant complications closely linked to aGVHD pathogenesis (96). However, validating candidate biomarkers requires large-scale, multicenter collaborative studies with standardized protocols for sample collection, processing, and storage. Additionally, the development of high-quality reagents and assays is essential to ensure the reliability and reproducibility of biomarker validation efforts (97). Future clinical trials should prioritize direct comparisons of different biomarkers under controlled conditions to establish standardized biomarker applications in clinical diagnostics and to improve GVHD risk assessment and management.

## 9 Therapeutic approaches for GVHD

### 9.1 Standard GVHD prophylaxis has traditionally relied on calcineurin inhibitor (CNI)-based regimens, most commonly Tacrolimus (Tac) or Cyclosporine A in combination with Methotrexate (MTX) or Mycophenolate Mofetil (MMF)

**Figure 1:**
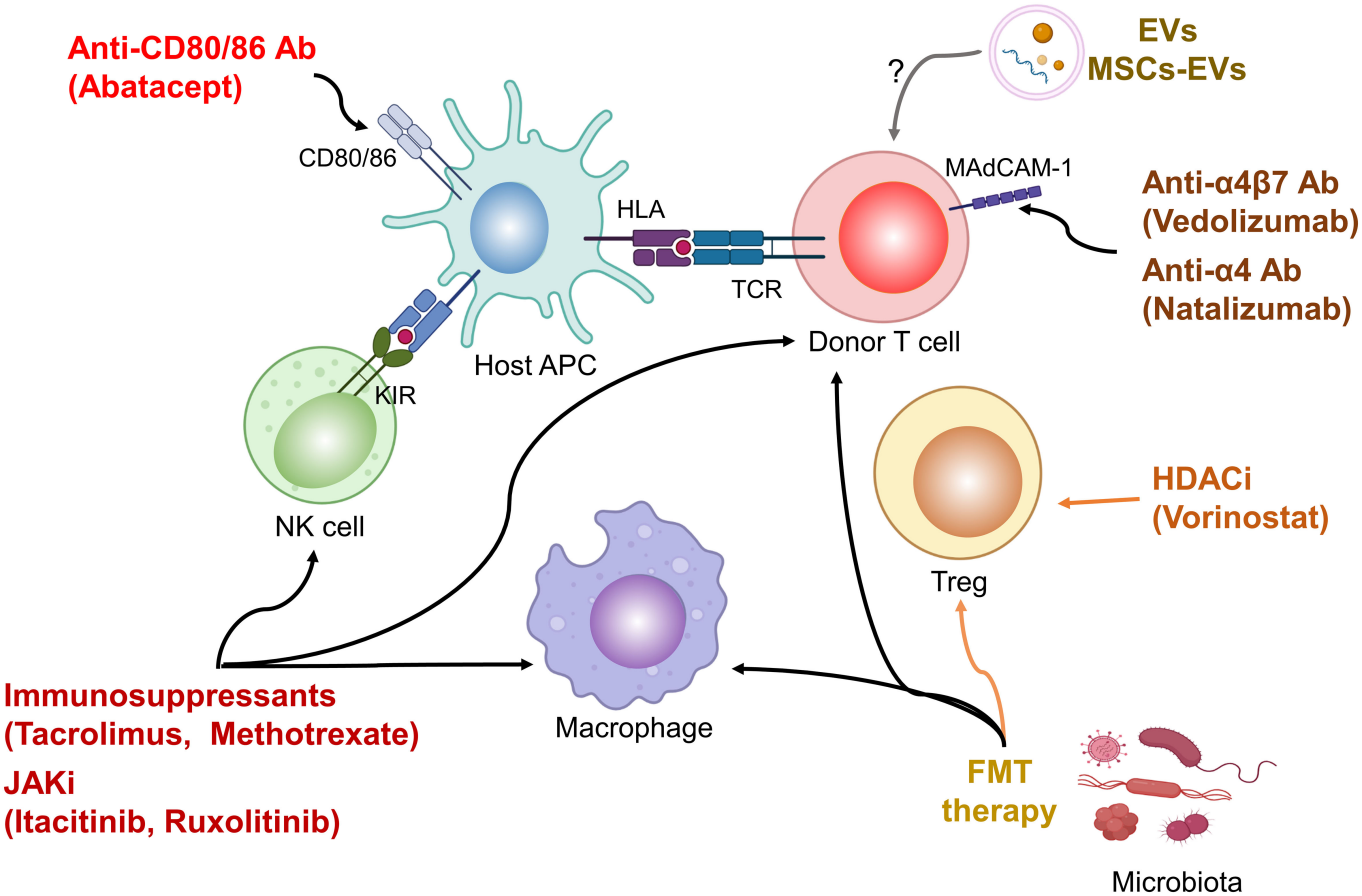
Therapeutic targets of graft-versus-host disease (GVHD). The diagram illustrates cellular interactions involved in GVHD pathogenesis and the mechanisms of various therapeutic interventions. Donor T cells are activated by Host Antigen-Presenting Cells (APCs) through HLA-TCR interaction and co-stimulation (CD80/86). Standard immunosuppressants (Tacrolimus, Methotrexate) and JAK inhibitors (Itacitinib) inhibit effects on NK cells, Macrophages, and Donor T cells. Abatacept (Anti-CD80/86 Ab) blocks T cell co-stimulation. HDAC inhibitors (Vorinostat) are utilized to modulate Regulatory T cell (Treg) function. Fecal Microbiota Transplantation (FMT) also regulates the gut microbiota, thereby influencing macrophage polarization and Treg induction. MSCs-derived Extracellular Vesicles (MSCs-EVs) represent a novel approach for potentially modulating T cell responses. The black lines indicate inhibition, while the orange lines indicate stimulation. Ruxolitinib is the current second-line standard of care for both SR-aGVHD and SR-cGVHD. APC, antigen-presenting cell; EVs, Extracellular Vesicles; FMT, Fecal Microbiota Transplantation; HDACi, Histone DeACetylase inhibitors; HLA, Human Leukocyte Antigen; JAKi, Janus Kinase inhibitors; MAdCAM-1, Mucosal Addressin Cell Adhesion Molecule-1; MSCs, Mesenchymal Stromal Stem Cells; NK cell, natural killer cell; TCR, T cell receptor; Treg, Regulatory T cells.

These approaches primarily function by suppressing alloreactive T-cell activation and proliferation. In recent years, post-transplant cyclophosphamide (PTCy) has emerged as a preferred and increasingly recommended platform (99), particularly in the setting of reduced-intensity conditioning (RIC) and donor transplantation. Mechanistically, PTCy selectively depletes proliferating alloreactive T cells while promoting immune tolerance (100). In parallel, Abatacept has received FDA approval for the prevention of aGVHD in unrelated donor HSCT by blocking T-cell co-stimulation. Numerous strategies are being explored for the prevention and treatment of GVHD, as shown in **Figure 1**. More recently, the randomized, multicenter BMT CTN 1703 Phase III trial demonstrated that a PTCy-based regimen (PTCy/tacrolimus/MMF) significantly improved relapse-free survival compared with standard tacrolimus–MTX in adult patients undergoing HSCT with reduced-intensity conditioning, supporting the evolving role of PTCy as a contemporary prophylactic backbone (98, 101, 102).

### 9.2 First-line treatment for aGVHD consists of systemic, topical, or non-absorbable oral corticosteroids

For steroid-refractory (SR) disease, Ruxolitinib is the second-line standard (103). For SR-cGVHD, four agents are currently approved: Ruxolitinib, Ibrutinib, Belumosudil, and Axatilimab (approved in August 2024) (104). Extracorporeal photopheresis (ECP) remains a vital non-pharmacological immunomodulatory option, particularly for cGVHD involving bronchiolitis obliterans syndrome.

Furthermore, targeted therapies are also being investigated, i.e., Axatilimab, Itacitinib, Abatacept, and Duplimab (33, 105–107). Itacitinib, a selective JAK1 inhibitor, has shown efficacy in preclinical models of aGVHD (108). Abatacept, a soluble fusion protein that blocks the co-stimulatory interaction between CD80/CD86 and CD28, is the first drug approved by the US Food and Drug Administration for the prevention of acute GVHD when combined with a Calcineurin inhibitor and MTX in patients undergoing unrelated donor HSCT (109). A case study also reported a successful response to dupilumab in a refractory case of atopic dermatitis-like chronic cutaneous GVHD (106). T-cell trafficking inhibitors like anti-α4β7 antibody (Vedolizumab) and anti-α4 antibody (Natalizumab) are used to target CD4+ T cells binding to mucosal vascular cell adhesion molecule 1 (MAdCAM-1) ligand, which is relevant for GI GVHD (33, 110, 111). Epigenetic-modifying drugs, such as histone deacetylase inhibitors (HDACi; Vorinostat), have been investigated for aGVHD prophylaxis to reduce inflammation and enhance regulatory T cells (112–114). Immunomodulators, such as α1-antitrypsin infusion, have been tested for steroid-resistant aGVHD (16, 110). Fecal microbiota transplantation (FMT) is another strategy being investigated for GI aGVHD (33). Notably, the Blood and Marrow Transplant Clinical Trials Network (BMT CTN) has recently launched a multi-center Phase 3 clinical trial (BMT CTN 2203, including the University of Michigan) testing Ruxolitinib in combination with tacrolimus/methotrexate for GVHD prevention. While Ruxolitinib is already approved for the treatment of cGVHD, its evaluation in the prophylactic paradigm focuses on JAK-STAT inhibition.

Since Le Blanc et al. first demonstrated the efficacy of mesenchymal stem cells infusion in treating a child with steroid-resistant aGVHD (115). Numerous studies have sought to validate the benefits of this therapy (116, 117). A meta-analysis of 13 studies involving 301 patients with steroid-resistant aGVHD showed an overall response rate of 68.1% (118). Early studies also highlighted the potential of bone marrow MSCs for the treatment of severe aGVHD; one multicenter study reported that patients with steroid-refractory aGVHD, who achieved a complete response, had a 53% two-year survival, compared to 16% among partial- and non-responders (119). Another meta-analysis of controlled trials demonstrated significant improvements in complete response rates and overall survival among patients with cGVHD treated with mesenchymal stromal/stem cells therapy compared with those treated with conventional therapy (120). In contrast, a systematic review by Fisher et al. analyzed results from 12 completed randomized clinical trials: seven to prevent GVHD and five to treat GVHD. The authors found significant deficiencies in the reported trials and concluded that the evidence does not support the beneficial effects of MSCs for treating or preventing GVHD (121). The use of MSCs to treat or prevent GVHD remains an open question requiring additional well-designed studies.

## 10 Conclusion

GVHD remains a significant challenge in allogeneic HSCT, impacting clinical outcomes. Advances in immunogenetics, RNA analysis, extracellular vesicles, microbiota research, proteomics, and emerging therapies have provided new insights into GVHD pathophysiology and potential therapeutic interventions. While substantial progress has been made, continued research efforts are necessary to refine diagnostic tools, develop targeted therapies, and improve long-term outcomes. A multidisciplinary approach integrating immunology, genetics, microbiology, and clinical medicine will be key to overcoming the complexities of GVHD and enhancing the widespread application of allogeneic HSCT for both malignant and non-malignant hematological conditions.
